# Do Three Different Passive Assessments of Quadriceps Spasticity Relate to the Functional Activity of Walking for Children Diagnosed with Cerebral Palsy?

**DOI:** 10.1155/2015/872015

**Published:** 2015-10-20

**Authors:** Hank White, Tim L. Uhl, Sam Augsburger

**Affiliations:** ^1^Motion Analysis Laboratory, Shriners Hospital for Children, Lexington, KY 40502, USA; ^2^Rehabilitation Sciences, College of Health Sciences, University of Kentucky, Lexington, KY 40536, USA

## Abstract

A stiff-knee gait pattern is frequently associated with several impairments including quadriceps spasticity in children diagnosed with cerebral palsy (CP). The relationship of clinical measures of quadriceps spasticity and the stiff-knee gait pattern in children diagnosed with CP has not been well established. Therefore, the purpose of this study was to determine the ability of clinical measures of quadriceps spasticity (modified Ashworth scale [MAS], Ely tests, and pendulum test) to categorize a stiff-knee gait pattern in children with CP. Children were categorized as having a stiff-knee gait pattern based on kinematic and EMG gait data. Results of a logistic regression model revealed that the only significant measure was A1 of the pendulum test. Discriminant analysis functions were used to predict group membership (stiff-knee, not stiff-knee gait pattern) for each measure. The A1 of the pendulum test demonstrated the highest classification accuracy and the highest sensitivity compared to the other measures. Therefore, a negative pendulum test (indicated by an A1 value of 45 degrees or more) is more useful for ruling out a stiff-knee gait pattern compared to the other clinical measures.

## 1. Introduction

The clinical presentation of cerebral palsy (CP) includes a broad spectrum of motor impairments of the neuromusculoskeletal systems such as joint contractures, decreased motor control, and muscle spasticity [1]. Stiff-knee gait pattern has been reported to be one of the most common gait abnormalities of children with a primary diagnosis of CP [2]. Quadriceps spasticity during late stance or early swing phase of the gait cycle has been proposed as the primary cause of a stiff-knee gait pattern [3, 4]. Decreased ankle power generation and/or decreased hip power generation could contribute to a stiff-knee gait pattern [5]. However, decreased hip and ankle power assessment requires utilization of motion analysis equipment that is not available to many clinicians. Clinically, the question remains: is the stiff-knee gait pattern due to quadriceps spasticity?

A review of clinical spasticity measures reported that most clinical scales (Modified Ashworth Scale and the Ely test) are subjective and their reliability and validity have not been thoroughly evaluated [6]. The Modified Ashworth Scale (MAS) is a six-point ordinal scale (0, 1, 1.5, 2, 3, and 4) based on subjective evaluation of a muscles resistance to passive movement perceived by the examiner with 0 indicating no resistance and 4 indicating that the joint is rigid [7–9]. Clinically 1+ is used to describe the third level of resistance. However a value of 1.5 can be assigned to the 1+ clinical score to maintain equal intervals [7]. Burridge et al. [10] recommend research assessing spasticity with more than one method and establishing relationships between clinical measures of spasticity and functional activities is needed. A nonsignificant relationship between the (MAS) and the knee motion during walking has been reported for children diagnosed with CP [11]. The Ely test can assess quadriceps flexibility, by measuring knee angle and pelvic rotation (Ely-F) or it can assess quadriceps spasticity, by measuring resistance with rapid passive knee flexion (Ely-S) [12, 13]. Two studies have reported that the Ely test is a useful predictor of quadriceps spasticity [12, 14]. However, a third study reported that a positive Ely test (as spasticity assessment) preoperatively did not influence postoperative results [15].

The pendulum test is an objective measure of quadriceps spasticity that has been reported in the literature for over 50 years [16–18]. During the pendulum test the subject's knee is passively extended by the examiner and then the leg is allowed to fall freely into flexion. If no upper motor neuron involvement is present, the knee typically demonstrates six or seven oscillations of flexion and extension, with each oscillation demonstrating a smaller arc of motion. If upper motor neuron involvement is present the knee motion is dramatically altered [19]. The A1 variable of the pendulum test is the maximal knee flexion measured during the first swing of the pendulum test. The value of A1 for children with CP was reported to be half the value compared to able-bodied children [20]. The pendulum test demonstrates moderate to high between-day reliability for children diagnosed with CP [20]. However, because the pendulum test requires instrumentation to measure knee motions, it is not routinely used clinically.

If a patient demonstrates a certain gait pattern, then certain clinical measures are performed to determine the cause of the gait deviation. However, the expected relationships between clinical measures and gait pattern are not always present, causing some to question whether a passive measure of an impairment can relate to the dynamic functional activity of walking [21]. Because quadriceps spasticity has been proposed as one cause of the stiff-knee gait pattern in children diagnosed with CP, the purpose of this study is to assess the ability of different measures (MAS, Ely tests, and pendulum test) to correctly categorize a stiff-knee gait pattern.

## 2. Materials and Methods

### 2.1. Participants

Procedures were approved by a local institutional review board and informed consent was obtained. Children previously diagnosed with spastic CP (diplegia or hemiplegia) and referred to our facility over a two-year period for clinical gait analysis were recruited for this prospective observational study (*n* = 277). Inclusion criteria for study participation were primary diagnosis of spastic CP and age range of 8 to 21 years and were classified using the Gross Motor Function Classification System (Table 1). Exclusion criteria were children who had undergone orthopaedic surgery in the twelve months prior to being seen in the motion analysis laboratory, children who had previously undergone a rectus femoris transfer, or children who could not correctly follow verbal instructions. A convenience sample of 68 children (40 boys, 28 girls) with a mean age of 11 years (SD 2 years) (range 8–18 years) participated in the study. Eight of the children had previously undergone a dorsal rhizotomy and two had a Baclofen pump in place.

### 2.2. Data Processing and Data Analysis

Data were collected in the same order for all children by one examiner. Kinematic and EMG data while walking were first collected using surface reflective markers and surface electrodes following the standard gait analysis protocol (Cleveland clinic marker set) [20]. Kinematic data were collected at 60 Hz using a Motion Analysis Corporation Real Time System (EvaRT 4.4.4) with eight Eagle digital cameras. OrthoTrak 6.24 software was used to reduce and plot kinematic data (Motion Analysis Corporation, Santa Rosa, CA). The raw data were filtered using a Butterworth filter at 6 Hz. Electromyographic data was collected at 1000 Hz using Noraxon's TeleMyo 900 system (Noraxon U.S.A. Inc., Scottsdale, AZ) with surface silver-silver chloride electrodes (ConMed Corporation, Utica, NY) [20]. After the walking trials were performed, each subject participated in the pendulum test lying comfortably in supine on a bench (seat to floor height 76 cm). Each subject was positioned so the posterior calf did not contact the bench when the knee was in maximum flexion. This was performed to ensure that the mat did not impede maximum knee flexion. The examiner positioned the subject's leg in maximum knee extension. To control the starting position of the test, the distance from the heel of the foot to the floor was measured for the first trial, and the same was used for all trials. Prior to each trial, the subject was instructed to let the leg swing freely once it is released by the examiner. One to three practice trials were performed prior to data collection. Data collection with the motion analysis system was initiated approximately one second before the examiner released the subject's foot. After the subject's leg came to rest, at least thirty seconds passed before the next trial was performed [20]. Three trials were collected on each limb.

The surface EMG and reflective markers were then removed and then a standard clinical examination was performed including three clinical tests of quadriceps spasticity. First, the MAS was performed using an ordinal scale (0, 1, 1.5, 2, 3, and 4) [22]. The MAS was performed with the subject in supine with head and trunk in neutral and both upper and lower extremities in comfortable resting position. The hip was flexed to less than 45 degrees and the knee was passively flexed and extended by the examiner at a rate of approximately 1 cycle per second to maintain a constant speed [7].

Next, the subject was placed in prone position. The Ely-S test (an assessment of quadriceps spasticity) was recorded as the presence/absence of resistance experienced by the examiner when performing prone knee flexion rapidly [14]. Then the Ely-F test (an assessment of quadriceps flexibility) was also performed in prone. The examiner stabilized the subject's pelvis by placing one hand on the sacrum, and then he slowly flexed the subject's knee. The Ely-F value was recorded numerically as the magnitude of knee flexion at which the pelvis began to rotate anteriorly was recorded [12].

### 2.3. Data Reduction

A subject's gait pattern was classified as stiff-knee if at least 4 out of 6 previously reported criteria of kinematic and electromyographic data were present [12, 23, 24] (see the Appendix). For children diagnosed with spastic diplegia the left and right leg were compared during pilot testing and found not to be different; therefore, one of the two legs was chosen by a flip of a coin and the left leg was used for this study. If a child was diagnosed with hemiplegia, then data from the involved side are reported. The A1 of the pendulum test was calculated in Microsoft Excel using methods previously described [20].

Statistical analyses were conducted using SPSS, V22.0. (Chicago, IL). In order to compare categorical and continuous clinical measures from those with and without stiff-knee gait, chi-square and two sample *t*-tests were used. Modified Ashworth Scores were treated as ordinal data and nonparametric test (Mann-Whitney *U* test) was used to compare all grades of MAS between the stiff-knee and not-stiff-knee groups. Logistic regression analyses were used to investigate the relationship of multiple independent measures (MAS [ordinal data], Ely-S [categorical data], Ely-F [continuous data], and pendulum test [continuous data]) and the binary outcome presence/absence of a stiff-gait pattern. Discriminant analysis was performed to assess the ability of the clinical measures to correctly identify stiff-knee gait pattern. For discriminant analysis MAS was recoded as dichotomous data (1/0; tone present/absent) where MAS zero was coded 0 and MAS 1 and above coded 1. Ely-S was also recoded as dichotomous data (1/0; spasticity present/absent).

## 3. Results

Thirty-one of the 68 (46%) children were classified positive for a stiff-knee gait pattern.  Table 2 contains the means and standard deviations for the dependent measures for the stiff-knee and not-stiff-knee groups.

All dependent and predictor measures were normally distributed (skewness < ±2). None of the 68 subjects demonstrated significant multiple measure outliers. Logistic regression model was significant (chi-square 23 with 4 degrees of freedom *p* < 0.001). The Cox and Snell *R* square value was 0.290 and the Nagelkerke *R* square values was 0.387 indicating 29–39% of variance was explained by the model. The only significant measure in the logistic regression model was A1 of the pendulum test [*p* < 0.05; *B* = −0.05; Exp(*B*) = 0.95] (Table 3). Discriminant analysis functions were used to predict group membership (stiff-knee, not-stiff-knee gait pattern) of subjects for each measure (Table 4). The A1 measure of the pendulum test demonstrated the highest classification accuracy (78%) (chi-square [1 df] = 19.27; Wilk's Lambda = 0.75, *p* < 0.001). For an A1 of 45 degrees, sensitivity was 87% and the specificity was 68% (Table 4).

## 4. Discussion

The impetus for this study came from interest in better understanding the relationship between measures of quadriceps spasticity and the stiff-knee gait pattern of children diagnosed with CP. Because quadriceps spasticity has been proposed as one cause of the stiff-knee gait pattern in children diagnosed with CP the purpose of this study was to assess the ability of measures (MAS, Ely tests, and pendulum test) to correctly categorize a stiff-knee gait pattern. Logistic regression analysis revealed that only the A1 of the pendulum test was a significant measure for correctly categorizing the children's gait pattern as stiff-knee or not. For each one-degree increase in the A1 the odds are decreased by 5% that the child will have a stiff-knee gait.

The regression analysis identified those clinical measures most related to the target problem (stiff-knee gait pattern). From the regression analysis, the greatest amount of variance of the knee gait patterns was explained by the A1 measure of the pendulum test. However, from a clinical perspective, this information is not very applicable. Clinically, it is more important to know the discriminant ability of a test, or how well a test can identify the target problem. Therefore, discriminant analysis was performed to assess the ability of the clinical measure to correctly identify stiff-knee gait pattern. The A1 measure demonstrated a higher overall accuracy to correctly classify the original groups as having a stiff- or not-stiff-knee gait pattern 77% compared to the other measures (MAS 69%, Ely-F 62%, and Ely-S 71%) (Table 4).

However, the overall accuracy of a test does not provide information regarding false positive and the false negative rate of a test [25, 26]. Therefore the sensitivity and specificity of a test are often reported as measures of the usefulness of a diagnostic test. The specificity of a test indicates the test's ability to correctly identify the absence of the target problem, and the sensitivity represents the correct identification of presence of the target problem [25, 26]. In our study, the A1 measure calculated from the pendulum test demonstrated the highest sensitivity (87%) compared to the other measures (MAS 48%, Ely-F 52%, and Ely-S 61%). The A1 measure demonstrated the lowest specificity (68%) compared to the other measures (MAS 87%, Ely-F 70%, and Ely-S 78%) (Table 4). Rarely a test demonstrates both high specificity and high sensitivity [25, 26]. A test with high sensitivity indicates that few false negative results occurred. When the test is negative, a test with high sensitivity is used to rule out the presence of the target problem. However, this does not provide information regarding whether the test results are positive [25, 26]. A test with high specificity is a test with few false positive results. When a test with high specificity is positive, that test is used to rule in the presence of the target problem [25, 26]. Therefore, a negative pendulum test (indicated by a large A1 value [greater than 45 degrees]) is potentially more useful for ruling out a stiff-knee gait pattern compared to the other measures.

A limitation of sensitivity and specificity is that one has to know if the target problem is truly present to calculate the sensitivity or specificity of a test. Sensitivity and specificity only indicate the probability of a correct test result (true positive, false positive, false negative, and true negative) [25, 26]. The sensitivity and specificity measures are not easily translated to an individual. Positive and negative likelihood ratios are ratios calculated from the specificity and sensitivity of a measure and can be easily translated to an individual [26, 27]. The positive likelihood ratio equals sensitivity/(1 − specificity). The negative likelihood ratio equals (1 − sensitivity)/specificity [26, 28]. There are several benefits to positive and negative likelihood ratios. These measures are less affected by the prevalence of the target problem, can be used with continuous measures, can be applied to individuals, and can refine clinical judgment [28].

By multiplying the pretest probability of a target problem by the likelihood ratio gives the posttests odds of the target problem [26, 28]. The A1 measure demonstrated a moderate negative likelihood ratio of 0.19 compared to the other measures small likelihood ratios (MAS 0.60, Ely-F 0.70, and Ely-S 0.50). Hypothetically, if a clinician was 50% confident a child did have a stiff-knee gait pattern, then the posttest probability of having a stiff-knee gait pattern for a negative result for each measure would be MAS (0.5*∗*0.6) = 0.30 (30%), Ely-F (0.5*∗*0.7) = 0.35 (35%), Ely-S (0.5*∗*0.5) = 0.25 (25%), and A1 (0.5*∗*0.19) = 0.10 (10%). These results indicate that a negative pendulum test would decrease the probability of a stiff-knee gait pattern more than a negative result for the other clinic measures. Therefore, a negative pendulum test (as evident by an increased magnitude of A1) decreased the pretest probability from 50% to the posttest probability of 10% that the child has a stiff-knee gait pattern. The positive likelihood ratio of the A1 measure (2.7) was similar to the other measures (MAS 3.6, Ely-F 1.7, and Ely-S 2.8), indicating that each measure demonstrates a similar increase in the posttest probability of identifying the target problem (stiff-knee gait) when a positive test is present.

Two possible explanations exist for the A1 measure of the pendulum test being the only significant measure in the logistic regression. First, four of the six criteria of a stiff-knee gait pattern and the A1 measure of the pendulum test are all measures of knee displacement. Second, the methodological difference between subjective clinical measures (Ely-F, Ely-S, and MAS) and the objective pendulum test are significant. During the pendulum test, a constant force (gravity) is applied and the knee motions are objectively measured by the motion analysis system. In contrast, the MAS and Ely-S test are subjective assessment performed at inconsistent knee flexion velocities. Therefore, the combination of a consistent application of force with a quantitative measurement of knee displacement motion, instead of subjective assessments, resulted in the pendulum test being the only significant measure to identify stiff-knee gait patterns.

Previous studies using the pendulum test and EMG data have only presented a visual example of EMG data [19]. This study is the first to report results of EMG activity for all subjects. EMG data revealed that 55 of 68 (81%) of the children demonstrated a burst of activity (amplitude two standard deviations above baseline), during the first swing of the pendulum test indicating that a stretch reflex of the quadriceps occurred during the pendulum test, supporting the premise that the pendulum test is in part a measure of quadriceps spasticity. In a study comparing the pendulum test awake and under anesthesia for children without disability and children diagnosed with CP, fewer differences were noted between the two groups while being under anesthesia [29]. The differences present under anesthesia were thought to be due to chronic changes in soft tissues. The authors concluded that the pendulum test is a quantitative measure of both the neural (stretch reflex) and nonneural (chronic changes in musculotendinous tissues) components of quadriceps spasticity [29]. Future studies using a more detailed analysis of the EMG activity could provide insight as to the amount the neural and nonneural components of quadriceps spasticity contribute to the different knee motions occurring during the pendulum test and walking.

A limitation of this study is that ten of the children had previously undergone spasticity reducing interventions (rhizotomy or baclofen pump). Due to the small sample size no detailed analyses were performed on this subset of children. However, consistent with previous study results [19], the eight children who had previously undergone a dorsal rhizotomy demonstrated a larger A1 (53 ± 29 degrees) compared to the children who had not received any type of spasticity reducing intervention (46 ± 22 degrees). Four of the eight children after rhizotomy demonstrated a burst of EMG activity during the first swing of the pendulum test. Conversely, the two children who had a Baclofen pump demonstrated a smaller A1 (26 ± 4 degrees) and both demonstrated a burst of EMG activity of the quadriceps during the first swing of the pendulum test. One goal of this clinical research is to develop an objective measure of spasticity [21]. These results indicate the potential of future studies to use the pendulum test as an objective spasticity measure to assess the effectiveness of different spasticity reducing interventions (medications and surgical).

Because the logistic regression model explained 29–39% of the variance of the gait patterns, there are other factors not accounted for by the model contributing to the stiff-knee gait pattern. Previous studies using computer modeling of children diagnosed with CP reported that the knee flexion velocity at toe-off had a larger influence on knee range of motion in swing than hip flexion velocity, knee angle, and hip angle at early swing phase [3]. Another modeling study proposed that the stiff-knee gait pattern in children diagnosed with CP could be caused by decreased force generation by the iliopsoas and gastrocnemius as well as by increased force generation of the quadriceps during the weight release phase of the gait cycle [5]. By implementing the pendulum test, clinicians can objectively and reliably identify if quadriceps spasticity is absent. If spasticity of the quadriceps is not present, as evident by 45 degrees or more of knee flexion during the first swing of the pendulum test, then other body structures or impairments (decrease force generation of the hip flexors and ankle plantar flexors) should be assessed for the cause of the stiff-knee gait pattern in a subject diagnosed with CP. Future studies assessing both the pendulum test results and the contribution of forces generated by the ankle and hip when walking could potentially develop a model explaining more of the variance of the knee gait pattern.

Presently the pendulum test requires the use of biomechanical instrumentation techniques (electronic goniometer or three-dimensional motion analysis system) to quantitatively measure the knee motion. Therefore, some clinicians may believe that the pendulum test is not a clinically useful tool. We acknowledge that this is a limitation of the pendulum test. However, a video camera or smart phone could record the knee motion during the pendulum test and then it could be played back and paused to estimate the value of A1. Therefore, future studies to assess the accuracy of visual analysis of the pendulum test are needed.

## 5. Conclusions

The purpose of this study was to assess the relationship of the different measures of quadriceps spasticity (MAS, Ely, and pendulum test) in the presence of a stiff-knee gait pattern, measured using motion analysis laboratory. Logistic regression analysis revealed that only the A1 of the pendulum test was a significant measure for correctly categorizing the children's gait pattern as stiff-knee or not. The A1 measure demonstrated a moderate negative likelihood ratio of 0.19. Hypothetically, if a clinician was 50% confident a child has a stiff-knee gait pattern. Multiplying the pretest probability of a target problem by the likelihood ratio gives the posttests odds of the target problem. Therefore, a negative pendulum test (as evident by an A1 greater than or equal to 45 degrees) decreases the pretest probability from 50% to the posttest probability of 10% that the child has a stiff-knee gait pattern. By implementing the pendulum test clinicians can objectively and reliably identify if quadriceps spasticity is absent. If spasticity of the quadriceps is not present, as evident by 45 degrees or more of knee flexion during the first swing of the pendulum test, then other body structures or impairments (decrease force generation of the hip flexors and ankle plantar flexors) should be assessed for the cause of the stiff-knee gait pattern in a subject diagnosed with CP.

## Figures and Tables

**Table 1:** Characteristics of subjects (*n* = 68).

Cerebral palsy	*n*	%
Diagnosis		
Spastic diplegia	55	81
Right hemiplegia	5	7
Left hemiplegia	8	12
GMFCS level		
I	29	43
II	24	35
III	14	21
IV	1	1
Assistive device		
None	53	78
Walker	9	13
Loft strand crutches	6	9

**Table 2:** Dependent and independent measures (^*∗*^ *p* < 0.05,  ^*∗∗*^ *p* < 0.01,  ^*∗∗∗*^ *p* < 0.001,  *p* values between stiff- and not-stiff-knee groups; ^#^statistical comparison includes all grades of Modified Ashworth Scores between stiff-knee and not-stiff-knee groups).

Measures	Stiff-knee (*n* = 31)	Not-stiff-knee (*n* = 37)
Criteria of knee gait pattern		
Knee flexion angular velocity at toe-off (degrees/sec), mean (SD)	120 (80)^*∗∗∗*^	263 (95)
Time to maximum knee flexion in swing (percent of swing phase), mean (SD)	55 (18)^*∗∗∗*^	43 (11)
Maximum swing phase knee flexion (degrees), mean (SD)	56 (13)	60 (6)
Total knee motion (degrees), mean (SD)	38 (14)^*∗∗∗*^	50 (13)
Inappropriate quadriceps EMG activity, *n* (%)	25 (81)	24 (65)
Toe drag, *n* (%)	11 (36)	1 (3)
Pendulum test		
A1 [max knee angle − start knee angle = amount of knee flexion occurring during first swing] (degrees) mean (SD)	34 (15)^*∗∗∗*^	57 (23)
Modified Ashworth Score *n* (%)		
0	16 (52)^*∗∗∗*#^	32 (87)
1	10 (32)	5 (13)
1.5	3 (10)	
2	2 (6)	
Ely-S (quadriceps spasticity) *n* (%)		
Yes	12 (39)^*∗*^	29 (78)
No	19 (61)^*∗*^	8 (22)
Ely-F (quadriceps flexibility) *n* (%)		
90 degrees or less	23 (74)	15 (40)
Greater than 90 degrees	8 (26)	22 (60)

**Table 3:** Logistic regression model.

Measures	*B*	SE	Wald	df	Sig. (*p* value)	Exp(*B*)	95% CI for Exp(*B*)	
MAS (Modified Ashworth Scale)	0.816	0.624	1.711	1	0.191	2.261	0.666	7.674
Ely-S test (quadriceps spasticity)	−0.268	0.734	0.134	1	0.715	0.765	0.181	3.223
Ely-F test (quadriceps flexibility)	−0.002	0.020	0.011	1	0.915	0.998	0.960	1.037
A1 (knee flexion during first swing of pendulum test)	−0.049	0.022	4.952	1	0.026	0.952	0.911	0.994
Constant	2.049	1.718	1.422	1	0.233	7.758		

**Table 4:** Sensitivity, specificity, and positive and negative likelihood ratios for clinical measures of spasticity (*n* = 68 subjects).

Spasticity measure	Overall accuracy	Sensitivity (95% CI)	Specificity (95% CI)	Positive likelihood ratio (95% CI)	Negative likelihood ratio (95% CI)
A1 of pendulum test	77%	0.87 (0.75–0.99)	0.68 (0.53–0.83)	2.69 (1.66–4.37)	0.19 (0.07–0.49)

MAS	69%	0.48 (0.31–0.66)	0.87 (0.76–0.98)	3.59 (1.47–8.76)	0.60 (0.42–0.86)

Ely-F	62%	0.52 (0.34–0.69)	0.70 (0.55–0.85)	1.74 (0.95–3.17)	0.69 (0.45–1.05)

Ely-S	71%	0.61 (0.44–0.78)	0.78 (0.65–0.91)	2.84 (1.45–5.57)	0.50 (0.31–0.80)

## Appendix

A subject's gait pattern was classified using the characteristics listed below. A participant gait could be rated from 0 (no characteristics present) to 6 (all characteristics present) for a stiff-knee gait pattern. For example, if a subject had any 4 characteristics present, then they would be rated as 4.

1. A delay in timing of maximum knee flexion in swing phase defined as two or more standard deviations above the normal value (as a percent of the swing phase of the gait cycle) [15].
2. Two or more standard deviations below the average normal value of maximum knee flexion occurring during swing phase [15].
3. Two or more standard deviations below the average normal value of total sagittal plane knee motion occurring throughout the gait cycle [15].
4. Two or more standard deviations below the average normal value of knee angular velocity at toe-off [15].
5. Impaired foot clearance considered present if the toe/foot was noted to drag on the ground (based on visual observation of “toe drag” during walking trials) during the swing phase of the gait cycle [30].
6. Inappropriate quadriceps activity was present if the dynamic EMG activity during the swing phase of the gait cycle was two or more standard deviations above the minimum activity during the stance phase of the gait cycle [31].
